# Association between serum 25-hydroxyvitamin D and physical performance measures in middle-aged and old Japanese men and women: The Unzen study

**DOI:** 10.1371/journal.pone.0261639

**Published:** 2021-12-23

**Authors:** Michiko Uchiyama, Satoshi Mizukami, Kazuhiko Arima, Takayuki Nishimura, Yoshihito Tomita, Yasuyo Abe, Natsumi Tanaka, Yuzo Honda, Hisashi Goto, Maiko Hasegawa, Youko Sou, Ritsu Tsujimoto, Mitsuo Kanagae, Makoto Osaki, Kiyoshi Aoyagi

**Affiliations:** 1 Department of Public Health, Nagasaki University Graduate School of Biomedical Sciences, Nagasaki, Japan; 2 Leading Medical Research Core Unit, Nagasaki University Graduate School of Biomedical Sciences, Nagasaki, Japan; 3 Department of Human Science, Faculty of Design, Kyushu University, Fukuoka, Japan; 4 School of Rehabilitation, Department of Physical Therapy, Tokyo Professional University of Health Science, Tokyo, Japan; 5 Department of Health and Nutrition Science, Nishikyusyu University, Kanzaki, Japan; 6 Department of Orthopedic Surgery, Nagasaki Rosai Hospital, Nagasaki, Japan; 7 Ken-Hoku Health Care Office, Nagasaki, Japan; 8 Medical Policy Division, Nagasaki Prefectural Government, Nagasaki, Japan; 9 National Health Insurance & Health Improvement Division, Nagasaki Prefectural Government, Nagasaki, Japan; 10 Department of Orthopedic Surgery, Nagasaki University Graduate School of Biomedical Sciences, Nagasaki, Japan; 11 Department of Rehabilitation, Nishi-Isahaya Hospital, Isahaya, Japan; Nanjing Medical University, CHINA

## Abstract

**Purpose:**

Regarding epidemiological studies, the role of vitamin D in musculoskeletal functionality (muscle weakness and physical performance) among elderly people is still controversial. The purpose of the present study was to investigate the associations between 25-hydroxyvitamin D [25(OH)D] and physical performance among community-dwelling middle-aged and old Japanese men and women.

**Methods:**

The subjects were community-dwelling 297 men and 415 women aged 50 years and over. Data on height (m) and weight (kg) were collected. Serum 25(OH)D, parathyroid hormone, calcium, and albumin levels were measured. Serum 25(OH)D was classified into deficiency group: < 20 ng/mL, insufficiency group: 20–30 ng/mL, and sufficiency group: ≧ 30 ng/mL. Physical performance was assessed by grip strength, chair stand time, and functional reach. Information on current smoking, alcohol drinking, regular exercise, any comorbidities (hypertension, heart disease, diabetes mellitus, lung disease, and stroke), and pain (lumbar and knee) was collected.

**Results:**

Vitamin D deficiency and insufficiency based on serum 25(OH)D levels were observed in 15.2% and 56.9% of men and 52.0% and 43.6% of women, respectively. In men, higher serum 25(OH)D levels were associated with better grip strength (p for trend = 0.003), chair stand time (p for trend = 0.042), and functional reach (p for trend <0.001). On the other hand, these parameters were not associated with serum 25(OH)D levels in women.

**Conclusion:**

A higher level of serum 25(OH)D was associated with better physical performance in men but not in women.

## Introduction

Vitamin D plays an important role in increasing the absorption of calcium and phosphate for the mineralization of the skeleton [1]. Vitamin D is produced in the skin by exposure to ultraviolet light or can be obtained orally which is then hydroxylated by the liver to the primary circulating form of 25-hydroxyvitamin D [25(OH)D], and further hydroxylated by the kidney to the active form of 1,25-dihydroxyvitamin D [1,25(OH)_2_D] [2]. Serum levels of 25(OH)D are a good marker of vitamin D levels in the body. Vitamin D deficiency causes secondary hyperparathyroidism, high bone turnover, bone loss, mineralization defects, and hip and other fractures [1]. Furthermore, vitamin D deficiency has been associated with increased risks of cancers, cardiovascular disease [2], and dementia [3].

In vitro studies have shown that the active form of vitamin D, calcitriol, drives cellular differentiation and proliferation by activating vitamin D receptors (VDR) in the nucleus of myocytes I [4, 5]. Endo et al. [6] have shown that VDR gene-deleted mice exhibited abnormal skeletal muscle development.

Regarding epidemiological studies, the role of vitamin D in musculoskeletal functionality (muscle weakness and physical performance) among elderly people is still controversial. Some studies reported the association between vitamin D deficiency and muscle weakness or poor physical performance [7–9]. The others did not show any associations [10–12].

The purpose of this study is to examine the associations of 25(OH)D with physical performance among community-dwelling people in Japan.

## Materials and methods

### Subjects

The subjects were community-dwelling men and women aged 50 years and over in Unzen city, Nagasaki Prefecture, Japan. The target population was approximately 13,000. Unzen City is located at latitude (N 32° 50’, E 130° 11’) and the residence area is almost located at the seaside. The main industries are agriculture, fishery, and tourism. This cross-sectional study used data obtained from periodic health examinations conducted from 2011 to 2013 (May to November; The Unzen Study). A total of 730 subjects (301 men and 429 women) participated in this study. Among those, participants with missing values were excluded, leaving 297 men [mean (standard deviation [SD]) age; 68.0 (8.3) years, range: 50–92 years] and 415 women [mean (SD) age; 67.7 (7.7) years, range: 50–89 years] for the final data analysis. In this study, 7 participants (1 man: 6 women) used vitamin D derivatives. Informed consent was obtained from all individual participants included in the study. This study was approved by the Ethics Committee of Nagasaki University Graduate School of Biomedical Sciences (11072739–2). All procedures performed were in accordance with the ethical standards of the institutional and/or national research committee and with the 1964 Helsinki declaration and its later amendments or comparable ethical standards.

### Measurements

Participants’ height was measured while shoeless using a wall-mounted stadiometer and weight was measured with the participant in light clothing, shoeless using a daily calibrated standard scale. Body mass index (BMI) was calculated as weight (kg)/height (m)^2^. Physical performance was assessed by handgrip strength, chair stand time, and functional reach. Grip strength was evaluated as an index of muscle strength in the upper limbs and was measured using a hydraulic hand dynamometer (Jamar hydraulic hand dynamometer; Lafayette Instrument Company, Inc., Lafayette, IN, US). Chair stand time was evaluated as an index of muscle strength in the lower limbs. It was quantified as the time taken to stand up from a standard chair and sit down five times, without the assistance of their arms. Functional reach was evaluated as an index of balance and was quantified as the difference between the initial point (standing comfortably upright, facing forward, hand in a fist, with the arm extended) and the reaching point (reaching forward as far as possible) without stepping or losing balance. All physical performance measures were performed twice and their excellent values were analyzed. When the test was conducted only once, the value was adopted.

### Questionnaire

Information on current smoking (yes/no), alcohol drinking (≧ 40 g/day in men and ≧ 20 g/day in women), regular exercise (at least 30 min twice per week) was collected by interview. Participants were asked if they had any comorbidities (hypertension, heart disease, diabetes mellitus, lung disease, and stroke) and pain (lumbar and knee). Comorbidity was defined as per diagnosis by a physician.

### Blood data

Fasting blood samples were collected, and 25(OH)D, parathyroid hormone (PTH), calcium, and albumin levels were measured. Serum 25(OH)D was measured by chemiluminescence enzyme immunoassay (CLEIA) and PTH by electrochemiluminescence immunoassay (ECLIA). Serum 25(OH)D levels ≥ 30 ng/mL, 20–30 ng/mL, and < 20 ng/mL were defined as vitamin D sufficiency, insufficiency, and deficiency, respectively.

### Statistical analysis

The student’s t-test was used to compare continuous variables and the chi- squared test to compare categorical variables between men and women. Simple correlation analysis and simple regression analysis were performed to examine the correlation between serum 25(OH)D and physical performance measures (grip strength, chair stand time, and functional reach). Differences in means by vitamin D status (deficiency, insufficiency, and sufficiency) were examined using one-way analysis of variance (ANOVA) and linear regression analysis. Analysis of covariance (ANCOVA) and multiple linear regression analysis were used to compare serum 25(OH)D and physical performance measures adjusting for age, BMI, PTH, albumin, calcium, current smoking, alcohol drinking, exercise, comorbidities (hypertension, heart disease, diabetes mellitus, lung disease, and stroke) and pain (lumbar and knee). A p-value of less than 0.05 was considered significant. The data were analyzed using IBM SPSS statistics version 25 (Armonk, NY, US).

## Results

Table 1 shows the characteristics of the participants. Serum 25(OH)D levels in men (25.9±5.8 ng/mL) were significantly higher compared to women (20.1±5.5 ng/mL) (p<0.001). The proportions of subjects with vitamin D deficiency and insufficiency were 15.2% and 56.9% in men and 52.0% and 43.6% in women, respectively. The prevalence of vitamin D deficiency was significantly higher in women than that in men.

**Table 1:** Characteristics of the participants.

Variable	Men (n = 297)	Women (n = 415)	p-value
Age (years)	68.0±8.3	67.7±7.7	.639
Height (cm)	163.3±6.6	151.3±5.7	<.001
Weight (kg)	62.4±9.9	50.6±7.9	<.001
Body mass index (kg/m^2^)	23.3±2.9	22.1±3.1	<.001
Serum			
25(OH)D (ng/mL)	25.9±5.8	20.1±5.5	<.001
Parathyroid hormone (pg/mL)	37.7±16.2	41.0±17.2	.010
Albumin (g/mL)	4.3±0.2	4.3±0.2	.999
Calcium (mg/mL)	9.2±0.4	9.2±0.3	.174
Physical performance measures			
Grip strength (kg)	37.1±8.6	24.4±5.3	<.001
Chair stand time (sec)	7.2±2.8	7.3±2.2	.891
Functional reach (cm)	35.8±8.2	33.8±7.1	<.001
	n (%)	n (%)	
Classification of 25(OH)D			<.001
deficiency	45 (15.2)	216 (52.0)	
insufficiency	169 (56.9)	181 (43.6)	
sufficiency	83 (27.9)	18 (4.3)	
Comorbidities			
Hypertension	131 (44.1)	169 (40.7)	.367
Heart disease	27 (9.1)	22 (5.3)	.049
Diabetes mellitus	35 (11.8)	13 (3.1)	<.001
Lung disease	13 (4.4)	12 (2.9)	.288
Stroke	14 (4.7)	15 (3.6)	.464
Lumbar pain	162 (54.5)	248 (59.8)	.165
Knee pain	120 (40.4)	167 (40.2)	.965
Current smoking	51 (17.2)	5 (1.2)	<.001
Alcohol drinking	192 (64.6)	51 (12.3)	<.001
Exercise	91 (30.6)	146 (35.2)	.205

Data are shown as means±standard deviation or n (%).

25(OH)D: 25-hydroxyvitamin D.

Student’s t-test for continuous variables.

Chi-square test for categorical variables.

**Fig 1:**
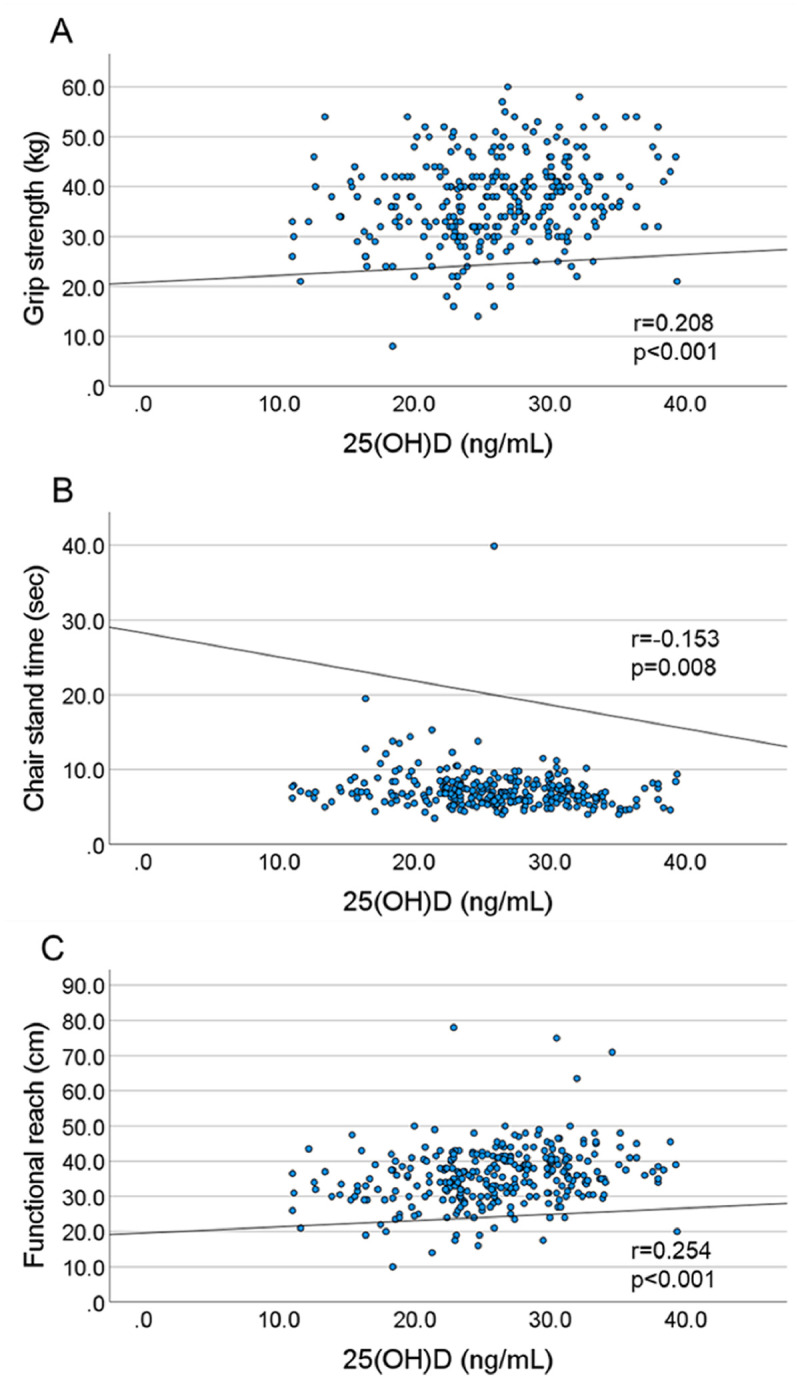
The scatter plot between serum 25(OH)D and physical performance measures in men. (A) Correlation between serum 25(OH)D and grip strength (r = 0.208, p<0.001). (B) Correlation between serum 25(OH)D and chair stand time (r = -0.153, p = 0.008). (C) Correlation between serum 25(OH)D and functional reach (r = 0.254, p<0.001).

**Fig 2:**
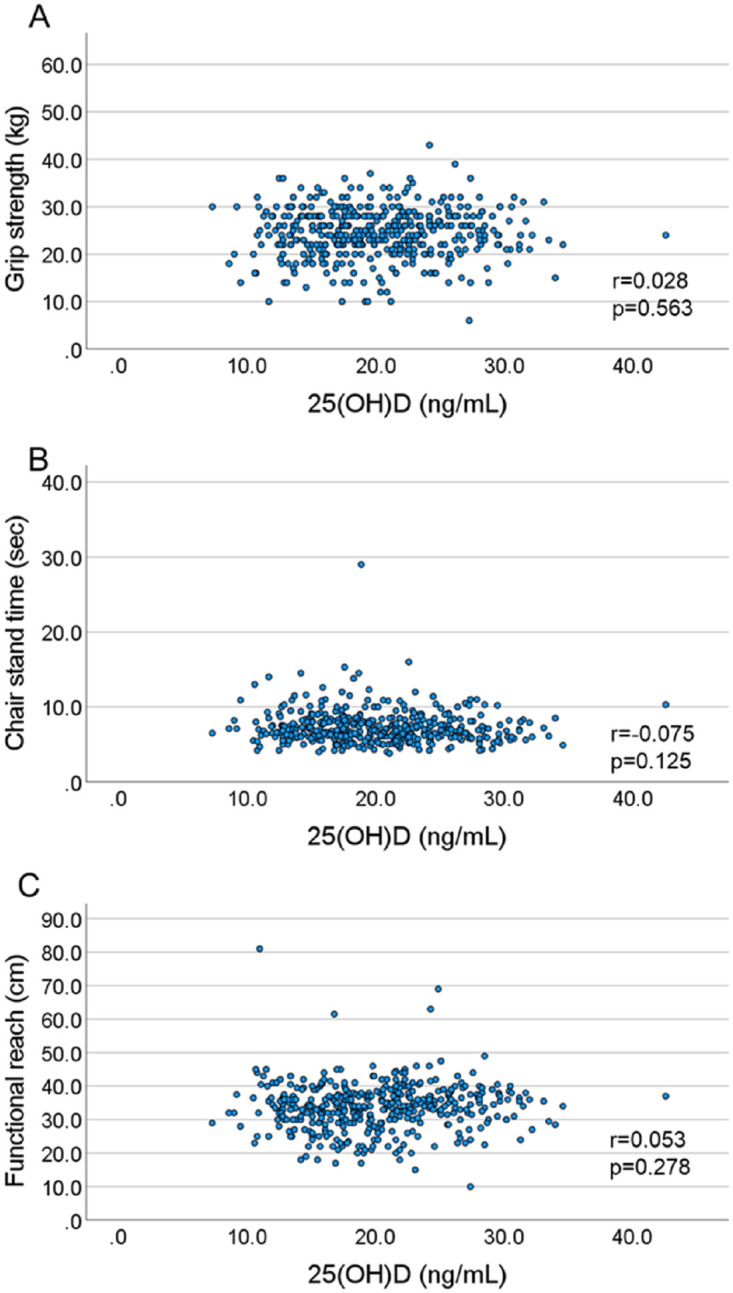
The scatter plot between serum 25(OH)D and physical performance measures in women. (A) Correlation between serum 25(OH)D and grip strength in women (r = 0.028, p = 0.563). (B) Correlation between serum 25(OH)D and chair stand time in women (r = -0.075, p = 0.125). (C) Correlation between serum 25(OH)D and functional reach in women (r = 0.053, p = 0.278).

Figs 1 and 2 show the scatter plots between serum 25(OH)D and physical performance measures among middle-aged and old Japanese men and women. The correlation coefficients between serum 25(OH)D and physical performances (grip strength, chair stand time, and functional reach) were 0.208 (p<0.001), -0.153 (p = 0.008), and 0.254 (p<0.001) among men, respectively (Fig 1). Alternatively, there were no significant correlations between serum 25(OH)D or any physical performance measure among women (Fig 2).

Table 2 shows the means (SDs) of physical performance measures according to serum 25(OH)D levels. In men, all physical performance measures were significantly associated with serum 25(OH)D levels (grip strength, chair stand time, and functional reach: p for trend = 0.002, 0.006, and <0.001, respectively). In women, chair stand time (p for trend = 0.022) and functional reach (p for trend = 0.048) were significantly associated with serum 25(OH)D levels, whereas grip strength was not associated.

**Table 2:** Means (standard deviations) of physical performance measures according to 25-hydroxyvitamin D [25(OH)D] levels.

Variable	Deficiency	Insufficiency	Sufficiency	p for trend
Men	(n = 45)	(n = 169)	(n = 83)	
Grip strength (kg)	34.8±8.3	36.5±8.8	39.4±7.9	.002
Chair stand time (sec)	8.2±2.9	7.2±3.1	6.7±1.5	.006
Functional reach (cm)	32.0±7.3	35.5±7.8	38.5±8.5	<.001
Women	(n = 216)	(n = 181)	(n = 18)	
Grip strength (kg)	24.3±5.3	24.6±5.4	24.1±4.7	.746
Chair stand time (sec)	7.5±2.5	7.0±1.8	6.9±1.4	.022
Functional reach (cm)	33.0±7.3	34.7±7.0	33.8±4.5	.048

One-way analysis of variance (ANOVA) and linear regression analysis.

Table 3 shows the adjusted means (standard errors) for physical performance measures according to 25(OH)D levels. In men, all physical performance measures (grip strength, chair stand time, and functional reach) were significantly associated with serum 25(OH)D levels after adjusting for covariates (age, BMI, PTH, albumin, calcium, current smoking, alcohol drinking, exercise, hypertension, heart disease, diabetes mellitus, lung disease, stroke, lumbar pain, and knee pain). Higher serum 25(OH)D levels were associated with better physical functioning in grip strength (p for trend = 0.003), chair stand time (p for trend = 0.042), and functional reach (p for trend < 0.001). On the other hand, grip strength, chair stand time, and functional reach were not associated with serum 25(OH)D levels in women (p for trend = 0.825, 0.085, and 0.368, respectively).

**Table 3:** Adjusted means (standard error) of physical performance measures according to 25-hydroxyvitamin D [25(OH)D] levels.

Variable	Deficiency	Insufficiency	Sufficiency	p for trend
Men	(n = 45)	(n = 169)	(n = 83)	
Grip strength (kg)				
age adjusted	34.8 (1.1)	36.8 (0.6)	38.7 (0.8)	.003
model 1	35.0 (1.1)	36.8 (0.6)	38.6 (0.8)	.007
model 2	34.7 (1.1)	36.9 (0.5)	38.7 (0.8)	.003
Chair stand time (sec)				
age adjusted	8.2 (0.4)	7.2 (0.2)	6.9 (0.3)	.012
model 1	8.1 (0.4)	7.1 (0.2)	7.0 (0.3)	.050
model 2	8.1 (0.4)	7.1 (0.2)	7.0 (0.3)	.042
Functional reach (cm)				
age adjusted	32.0 (1.1)	35.8 (0.6)	38.0 (0.8)	<.001
model 1	31.7 (1.1)	35.8 (0.6)	38.0 (0.8)	<.001
model 2	31.7 (1.1)	35.9 (0.6)	38.0 (0.8)	<.001
Women	(n = 216)	(n = 181)	(n = 18)	
Grip strength (kg)				
age adjusted	24.4 (0.3)	24.4 (0.4)	24.0 (1.1)	.846
model 1	24.4 (0.3)	24.4 (0.4)	24.3 (1.1)	.902
model 2	24.5 (0.3)	24.3 (0.4)	24.4 (1.1)	.825
Chair stand time (sec)				
age adjusted	7.5 (0.1)	7.1 (0.2)	7.0 (0.5)	.049
model 1	7.5 (0.1)	7.1 (0.2)	7.0 (0.5)	.061
model 2	7.4 (0.1)	7.1 (0.2)	7.0 (0.5)	.085
Functional reach (cm)				
age adjusted	33.2 (0.5)	34.5 (0.5)	33.7 (1.6)	.099
model 1	33.3 (0.5)	34.5 (0.5)	33.2 (1.5)	.234
model 2	33.4 (0.5)	34.3 (0.5)	33.2 (1.5)	.368

Analysis of covariance (ANCOVA) and multiple linear regression analysis.

model 1: adjusted for age, BMI, parathyroid hormone, albumin, calcium, current smoking, alcohol drinking, and exercise.

model 2: adjusted for age, BMI, parathyroid hormone, albumin, calcium, current smoking, alcohol drinking and exercise, hypertension, heart disease, diabetes mellitus, lung disease, stroke, lumbar pain, and knee pain.

## Discussion

We showed that serum 25(OH)D was associated with grip strength, chair stand time, and functional reach in men, after adjusting for age, BMI, and the other covariates. Men with 25(OH)D level < 20 ng/mL had poorer physical performances compared to those with level ≧ 30 ng/mL. In CHIANTI Study, vitamin D levels were significantly associated with short physical performance battery (SPPB) score in men and handgrip strength in both men and women [7]. In the Health, Aging, and Body Composition Study, participants with 25(OH)D < 50 nmol/L had significantly poorer physical performance and slower gait speed, lower knee extensor, and grip strength than those with 25(OH)D ≧75 nmol/L [13]. These reports support our results observed in men.

On the other hand, we did not observe the association between serum 25(OH)D and physical performance in women. Sufficiency of vitamin D may be a possible explanation for these contradictory findings between the sexes. Prevalence of sufficiency (25(OH)D≧ 30 ng/ml) was 27.9% in men, whereas only 4.3% in women. Serum 25(OH)D concentrations tended to be lower in women than in men [14]. Women in the present study often used long sleeves, hats, and gloves, and were more likely to use sunscreen during outdoor activities. Several studies showed that serum 25(OH)D was not associated with physical performance among populations with a sufficiency rate of < 10% [11, 15]. When vitamin D sufficiency is very low, the effect of vitamin D on muscle strength and physical performance may not be measured. However, Iolascon et, al [16] reported that serum 25(OH)D value ≧ 30n g/mL sufficiency was 49.4% among a group of Italian postmenopausal women, and a significant association between serum 25(OH)D concentration and grip strength, knee extension muscle strength, and short physical performance battery (SPPB) score. If vitamin D sufficiency was high in women, it may have affected the results of this study.

Muscle mass has been reported to be greater in men than in women [17]. Muscle mass in women in our study may be lower than the threshold at which vitamin D asserts effects, contributing to the lack of an association of vitamin D with muscle strength and physical performance. Further study is needed to include muscle mass measurement.

Low serum 25(OH)D is prevalent among the elderly. In this study, the overall prevalence of vitamin D insufficiency and deficiency was 49.2% and 36.7%, respectively, and was higher in women than in men (vitamin D insufficiency: men, 56.9%; women, 43.6%; vitamin D deficiency: men, 15.2%; women, 52.0%). Another study in Japan reported that the overall prevalence of vitamin D insufficiency and deficiency was 81.3% and 1.2%, respectively, and was higher in women than in men (vitamin D insufficiency: men, 72.1%; women, 86.3%; vitamin D deficiency: men, 0.3%; women, 1.7%) [18]. Previous studies have suggested that vitamin D supplementation results in less severe functional limitations, fewer falls, and fractures [19]. Treatment of low serum 25(OH)D may improve quality of life among the elderly through the prevention of poor physical functioning.

Serum 25(OH)D has been reported to vary seasonally [20–23]. Our study was conducted from May to Nov. Nakamura et al. [23] reported that serum 25(OH)D concentrations in May-Nov were relatively high. The results of our study may reflect serum 25(OH)D during the time of the year when they are generally higher, possibly as a result of sun exposure.

Vitamin D3 signaling enhanced the effect of physical exercise and increased locomotive ability in mice [24]. Mice with inactivation of VDR in myocytes (mVDR) had a lower grip strength and slower running speed than controls [25]. Levels of 25(OH)D3 were inversely associated with HbA1c levels, and for patients with diabetes, were lower 25(OH)D than controls [26]. Higher HbA1c was related to weaker grip strength in patients with diabetes [27, 28]. Since the prevalence of diabetes was higher among men in this study, some men with diabetes with vitamin D deficiency might induce weaker muscle strength and poor physical performance.

Some previous studies reported an association between vitamin D deficiency and sarcopenia (low muscle mass, weak muscle strength, and poor physical performance) [29–31], while some others did not [32, 33]. Kim et al. [31] reported that vitamin D levels were significantly lower in the sarcopenia group among both men and women. Conversely, there was no difference in the serum vitamin D level between participants with or without sarcopenia among both men and women [32, 33]. Li et al. [34] reported that their sarcopenia group had significantly lower 25(OH)D than the non-sarcopenia group among men, but not women, which was similar to our results. Women in the present study had lower serum 25(OH)D than men; thus relationships might be estimated for different ranges of serum 25(OH)D for men and women [35].

This study has several limitations. First, since this is a cross-sectional study, we could not provide a causal relationship between vitamin D and physical performance. Second, there is a possibility of selection bias because our subjects were periodic health examination participants. Third, we could not assess protein intake, calcium/vitamin supplementation, hours of sun exposure, or renal function which may affect vitamin D metabolism and cognitive function which may affect outdoor physical activity.

## Conclusion

The present study showed that a higher level of serum 25(OH)D was associated with better physical performance in men but not women. These differences between the sexes may be due to the sufficiency of vitamin D or muscle mass. Further studies are needed to explore the association between vitamin D and physical performance.

## Supporting information

S1 Data (CSV)

S1 Questionnaire (TIF)

S2 Questionnaire (TIF)

S3 Questionnaire (TIF)

S4 Questionnaire (TIF)
