# Supplementary figures and images for: Association between serum 25-hydroxyvitamin D and physical performance measures in middle-aged and old Japanese men and women: The Unzen study
Source: PLoS One. 2021 Dec 23;16(12):e0261639. doi: 10.1371/journal.pone.0261639 (PMC8699688; doi:10.1371/journal.pone.0261639)

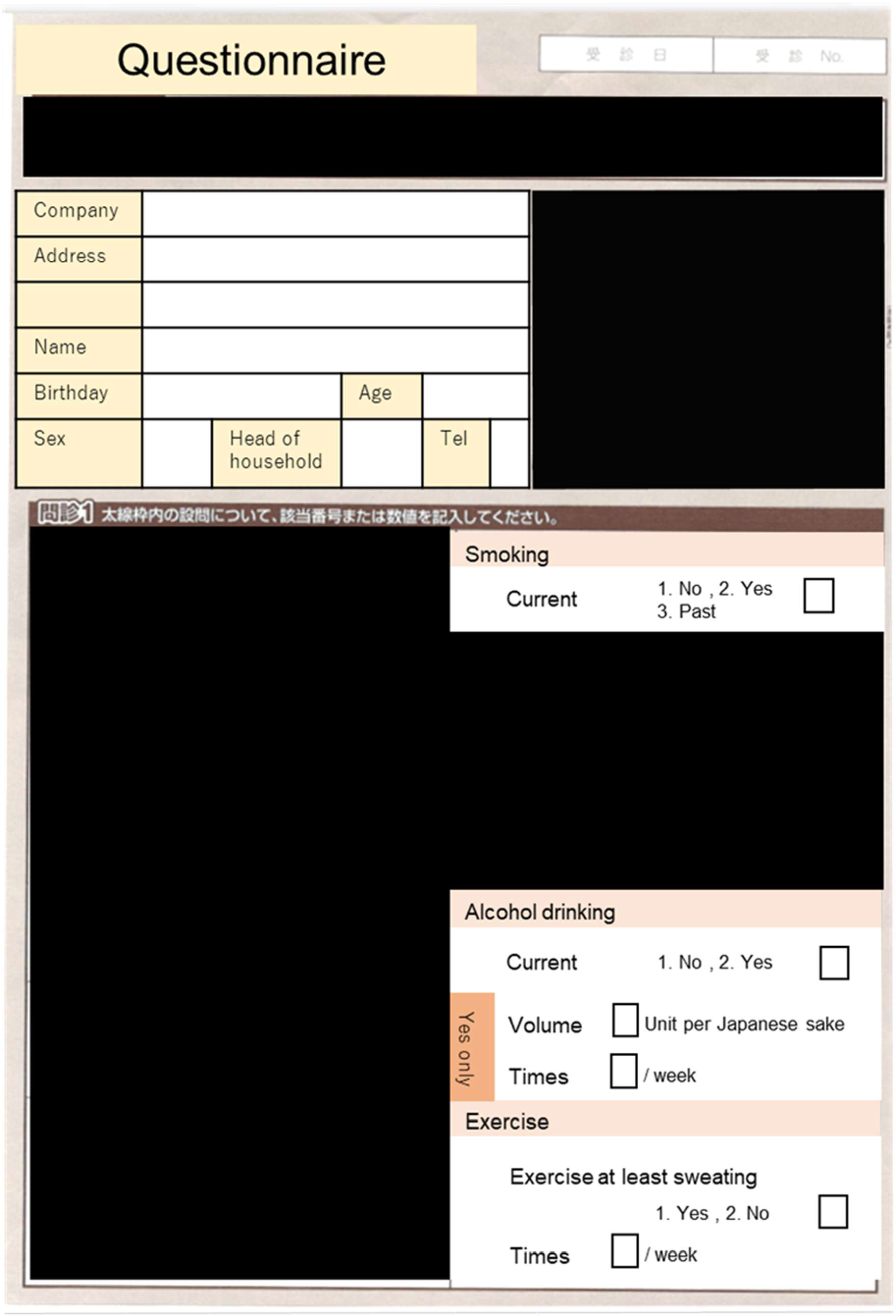

Supplement: S1 Questionnaire — (TIF) [file pone.0261639.s002.tif]

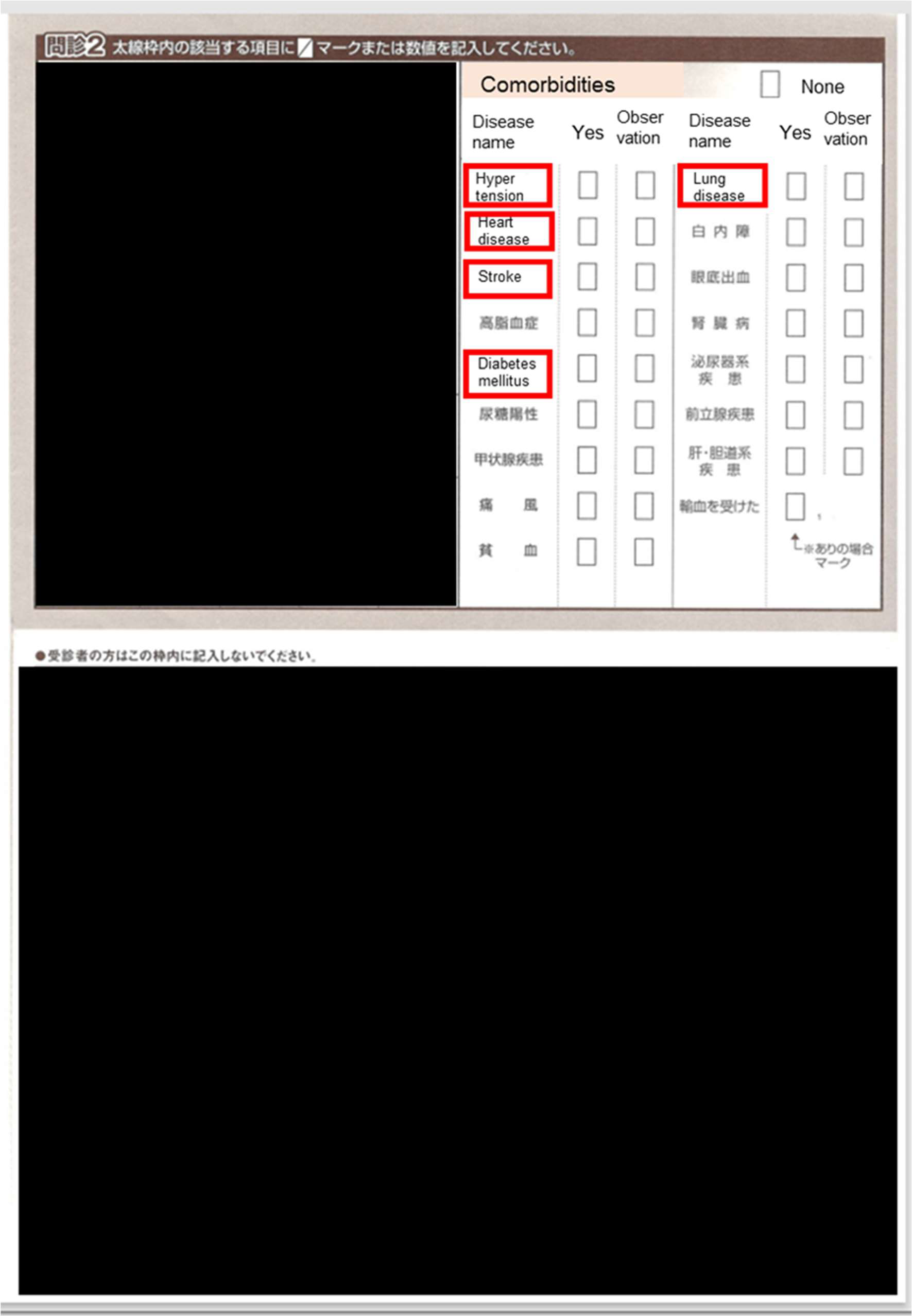

Supplement: S2 Questionnaire — (TIF) [file pone.0261639.s003.tif]

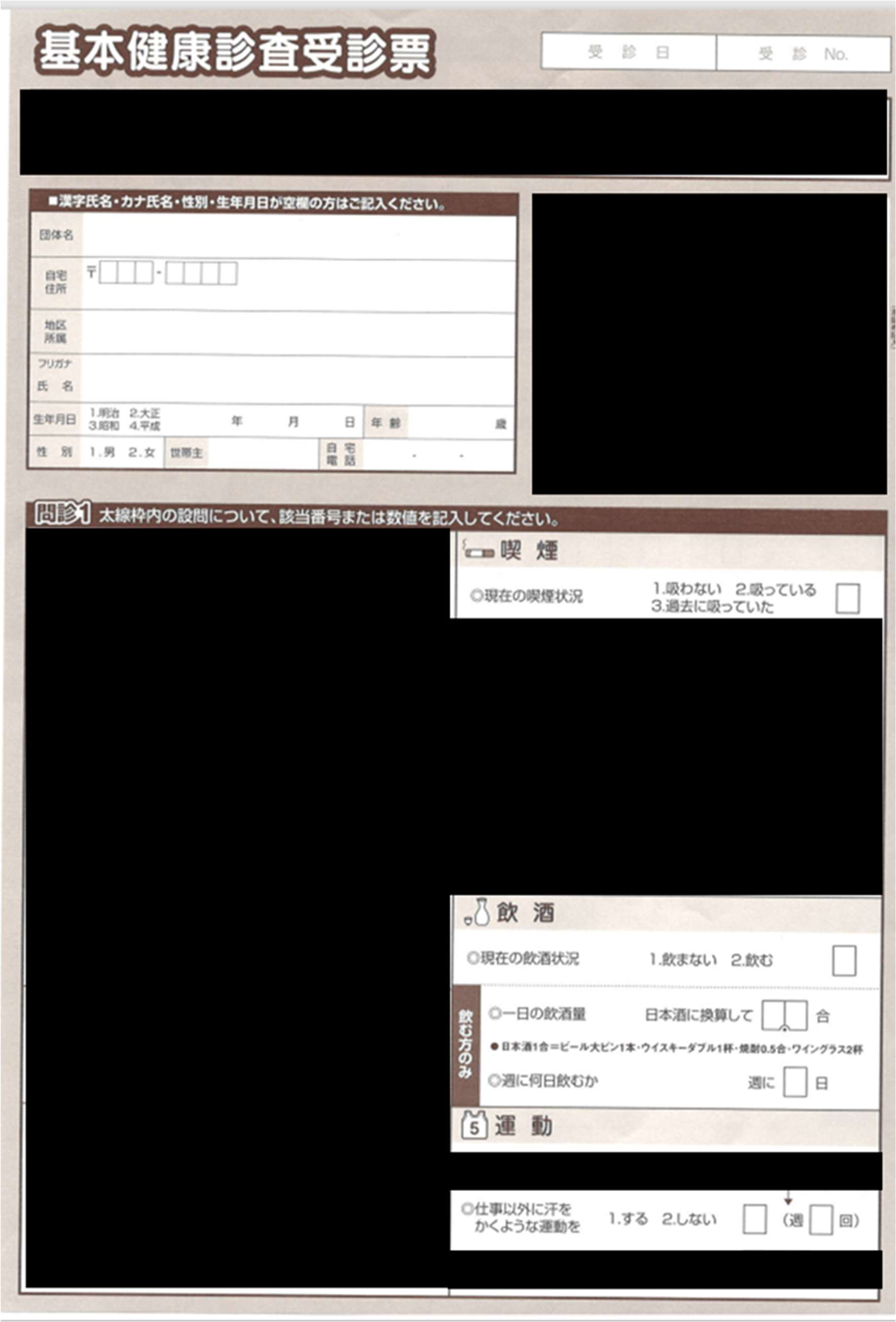

Supplement: S3 Questionnaire — (TIF) [file pone.0261639.s004.tif]

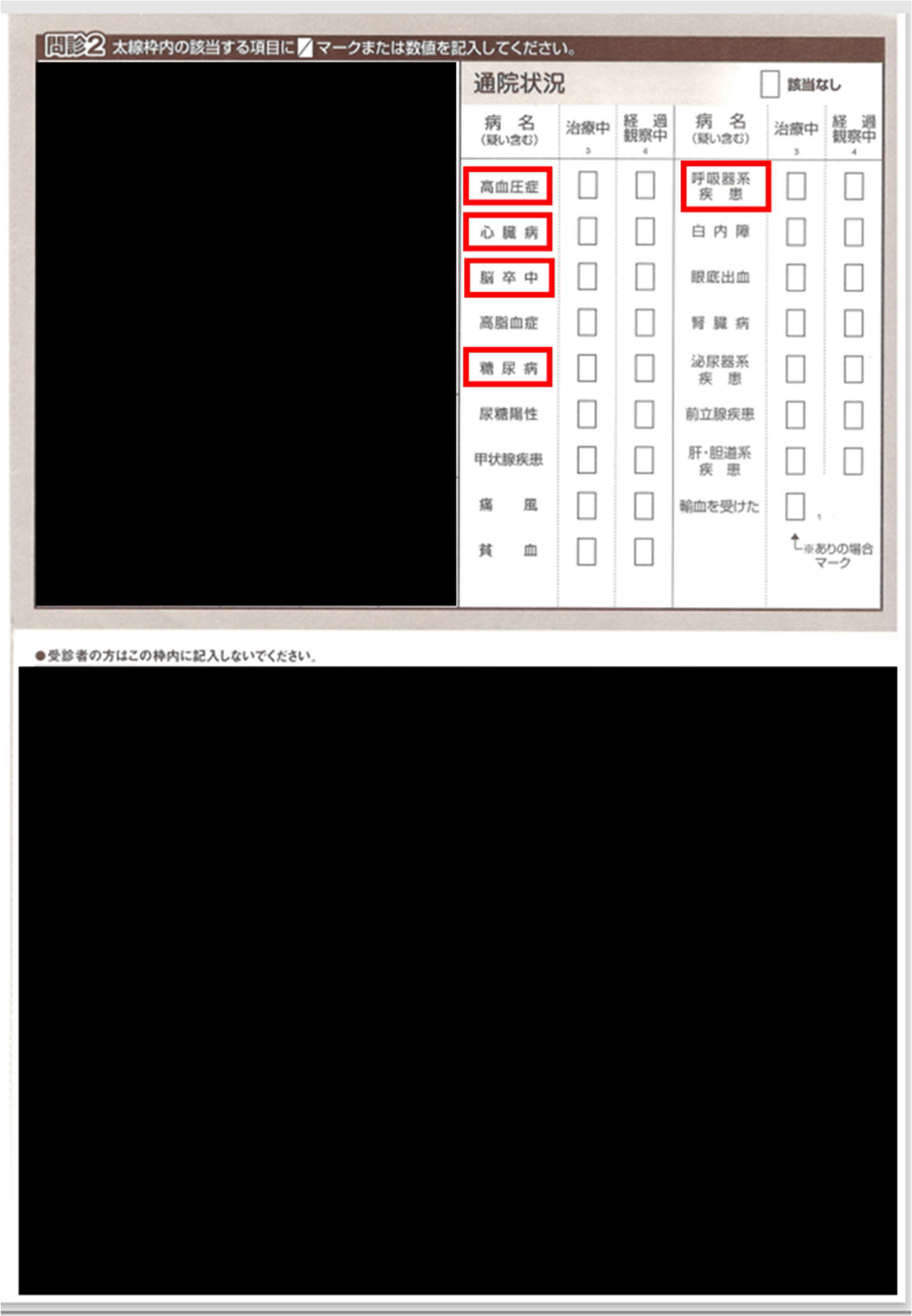

Supplement: S4 Questionnaire — (TIF) [file pone.0261639.s005.tif]
